# Vaccine and therapeutic agents against the respiratory syncytial virus: resolved and unresolved issue

**DOI:** 10.1002/mco2.70016

**Published:** 2024-11-21

**Authors:** Qianqian Li, Huan Li, Zhihua Li, Youchun Wang

**Affiliations:** ^1^ Institute of Medical Biology Chinese Academy of Medical Sciences and Peking Union Medical College Kunming China; ^2^ State Key Laboratory of Respiratory Health and Multimorbidity Institute of Medical Biology Chinese Academy of Medical Sciences and Peking Union Medical College Kunming China; ^3^ Key Laboratory of Pathogen Infection Prevention and Control (Peking Union Medical College) Ministry of Education Institute of Medical Biology Chinese Academy of Medical Sciences and Peking Union Medical College Kunming China

**Keywords:** nirsevimab, palivizumab, prefusion protein, respiratory syncytial virus, structure‐based vaccine, ziresovir

## Abstract

Respiratory syncytial virus (RSV) is a predominant pathogen responsible for respiratory tract infections among infants, the elderly, and immunocompromised individuals. In recent years, significant progress has been made in innovative vaccines and therapeutic agents targeting RSV. Nevertheless, numerous challenges and bottlenecks persist in the prevention and treatment of RSV infections. This review will provide an overview of the resolved and unresolved issues surrounding the development of vaccines and therapeutic agents against RSV. As of September 2024, three RSV vaccines against acute lower respiratory infections (ALRI) have been approved globally. Additionally, there have been notable progress in the realm of passive immunoprophylactic antibodies, with the monoclonal antibody nirsevimab receiving regulatory approval for the prevention of RSV infections in infants. Furthermore, a variety of RSV therapeutic agents are currently under clinical investigation, with the potential to yield breakthrough advancements in the foreseeable future. This review delineates the advancements and challenges faced in vaccines and therapeutic agents targeting RSV. It aims to provide insights that will guide the development of effective preventive and control measures for RSV.

## Graphical abstract

Breakthroughs have been made in the prevention and treatment of respiratory syncytial virus (RSV). Three RSV vaccines, two RSV monoclonal antibodies, and one RSV therapeutic agent have been approved for marketing, and multiple other products are in the clinical study phase. But RSV vaccines, monoclonal antibodies, and therapeutic agents all still have several unresolved issues

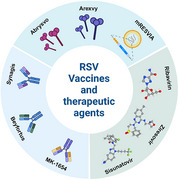

## 1 INTRODUCTION

Respiratory syncytial virus (RSV), classified within the Orthopneumovirus genus of the Pneumoviridae family, is an enveloped RNA virus that features a single‐stranded, negative‐sense genome.^1^ RSV infection is associated with increased mucus secretion, inflammatory responses, and airway constriction, establishing it as a pivotal etiological agent in the pathogenesis of acute lower respiratory infections (ALRI), particularly in the pediatric and geriatric populations.^2^ The susceptible age of RSV is distributed in a U‐shaped curve, with peak incidences in children under the age of 5 years and adults over the age of 65 years, typically peaking in late winter and early spring.^3^ In the pediatric population, RSV is identified as the principal etiologic agent precipitating hospitalization due to viral respiratory infections, with bronchiolitis, pneumonia, and tracheobronchitis being the predominant clinical manifestations of severe RSV infection. Every year, it causes approximately 33 million lower respiratory tract infections (LRTI), 3.6 million hospitalizations, and more than 100,000 deaths in children under 5 years old worldwide.^4^ In the elderly population over 65, RSV is a critical pathogen for pneumonia, with exacerbations of bronchiolitis, pneumonia, asthma, chronic obstructive pulmonary disease, or congestive heart failure being the main presentations of severe infection in this demographic.^5^ Annually, it leads to 336,000 hospitalizations and 14,000 in‐hospital deaths among the elderly worldwide.^6^ RSV has two subtypes, type A and type B, which are randomly cross‐circulated in different years.^7^ RSV is widely prevalent around the world, with local outbreaks.^8^ People of different ages are widely susceptible to the virus and are very prone to repeated infection.^3^ The hospitalization rate and mortality rate caused by RSV‐ALRI remain high, and effective prevention and treatment methods are urgently needed.

The RSV genome is 15.2Kb long and contains 10 genes, with 3′ to 5′ being distributed as NS1‐NS2‐N‐P‐M‐SH‐G‐F‐M2‐L.^9^ These genes encode for 11 distinct proteins, including two nonstructural proteins and nine structural proteins. The RSV virus is enveloped by a phospholipid bilayer with attachment glycoprotein (G), fusion protein (F), and small hydrophobic protein (SH) embedded on the surface.^10^ Among them, G and F proteins are the primary antigenic proteins that stimulate the production of neutralizing antibodies in the host organism.^11^ G protein is primarily responsible for the adherence to host cells,^12^ while F protein plays a crucial role in viral fusion, budding, and dissemination.^13^, ^14^ Due to the high subtype specificity and hypervariability of the G protein, with the central conserved “CX3C” motif being the only neutralizing epitope, there are very few vaccines and antibodies designed against it.^15^, ^16^, ^17^ In contrast, F protein is highly conserved across the A and B subtypes of RSV, making it a primary target for vaccine and antibody development.^18^, ^19^ SH protein is a pentamer ion channel protein that participates in viral replication and can delay apoptosis of infected cells. The RNA‐dependent RNA polymerase complex (RdRp)^20^ is composed of a large polymerase subunit (L), a phosphoprotein polymerase cofactor (P), and a nucleoprotein (N). The M2 gene contains two overlapping open reading frames that produce the transcription elongation factor M2‐1 protein and the M2‐2 protein, which controls the transition from transcription to genome replication.^21^, ^22^ The nonstructural proteins NS1 and NS2 work in concert to inhibit apoptosis and interferon response of cells.^23^, ^24^

**FIGURE 1:**
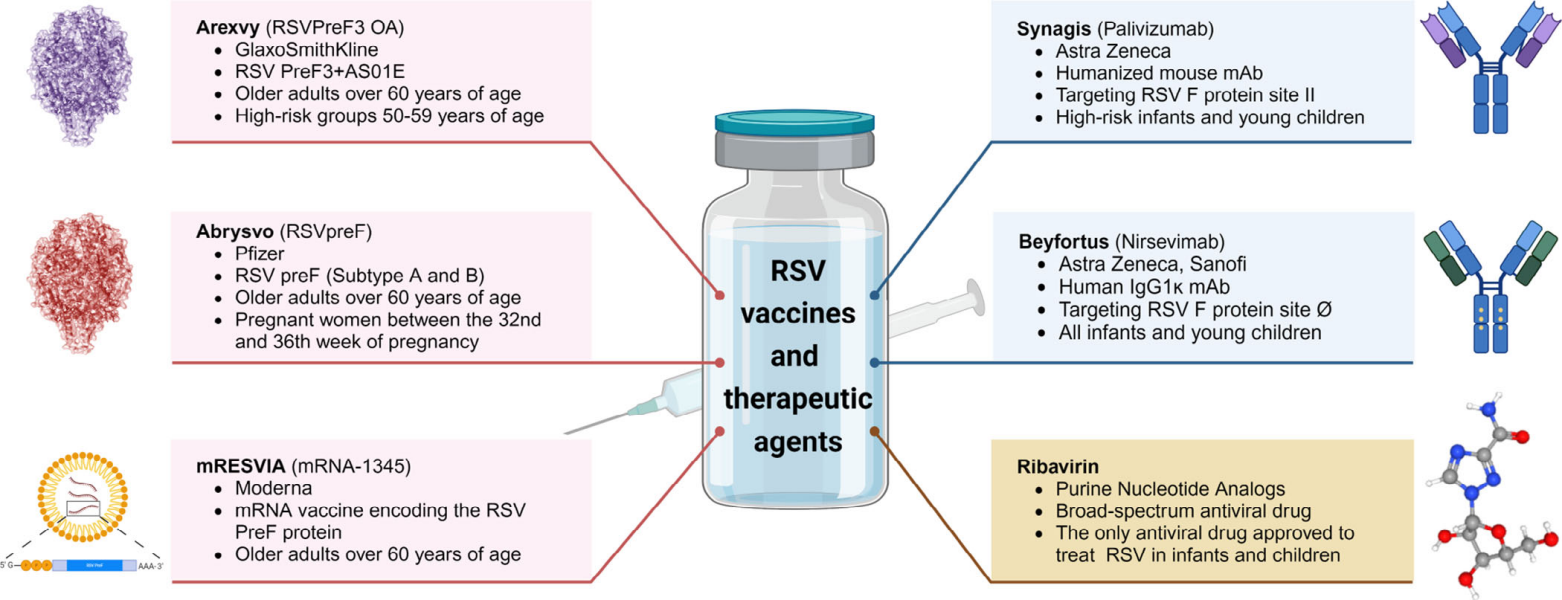
Approved RSV vaccines and therapeutic agents. Three RSV vaccines have been approved for marketing, Arexvy (RSVPreF3 OA), Abrysvo (RSVpreF), and mRESVIA (mRNA‐1345), are shown with pink lines and backgrounds. Two RSV mAbs have been approved for marketing, Synagis (Palivizumab) and Beyfortus (nirsevimab), and are shown with blue lines and backgrounds. Ribavirin is currently the only therapeutic agent approved for acute RSV infection and is shown with brown lines and backgrounds. Figure 1 is created using BioRender (https://www.biorender.com/).

Prevention and treatment of RSV infection represents an unmet medical need that has persisted over time. Since the discovery of the RSV, researchers have made numerous efforts and attempts to prevent and treat RSV infection (Figure 1), primarily focusing on vaccines, antibodies, and therapeutic drugs.^25^, ^26^, ^27^, ^28^ Vaccines can provide immunological protection to vulnerable populations, thereby reducing the risk of infection and curbing the incidence and transmission of the disease.^29^ GlaxoSmithKline (GSK)’s Arexvy vaccine^30^, ^31^ (for the elderly), Pfizer's Abrysvo vaccine^32^, ^33^ (for the elderly and pregnant women), and Moderna's mRESVIA vaccine^34^ (for the elderly) have all been approved for marketing and have shown excellent commercial performance, indicating the urgent demand for RSV vaccines. However, there are currently no vaccines available for infants and young children, making passive immunization with antibodies the only means of preventing RSV infection in this age group.^35^ Currently, the prophylactic antibodies available for passive immunization include Synagis^36^, ^37^, ^38^ (palivizumab, MedImmune) and Beyfortus^39^, ^40^ (nirsevimab, jointly developed by AstraZeneca and Sanofi), which are used to prevent RSV infection in infants and young children. To exacerbate the situation, there is currently no approved antiviral drug for clinical use, and treatment measures are mostly symptomatic supportive treatment. Early antiviral treatments mainly involved the use of broad‐spectrum antiviral drugs, such as ribavirin and recombinant human interferon alpha, but their efficacy remains controversial.^41^, ^42^

The review aims to summarize the current progress and challenge in the development of RSV vaccines, antibodies and therapeutic drugs.

## 2 RSV VACCINE

### 2.1 Foundation, consensus, and resolved issues of RSV vaccine

The successful commercialization of RSV vaccines is contingent upon advancements in structural biology, particularly the structural elucidation of the RSV F protein. Currently, there are a variety of RSV vaccines, with variations in vaccine types suitable for different demographic groups.^43^

#### 2.1.1 Structure‐based design of RSV vaccines

The F protein is highly conserved across the A and B subtypes of RSV and serves as a primary target for vaccine development. As a typical type I transmembrane protein comprising 574 amino acids, F protein exists as a trimer with a molecular weight of approximately 190 kDa. F protein exhibits two distinct conformational states, transitioning from the prefusion state (PreF) to the postfusion state (PostF) during the process of mediating membrane fusion.^14^ The conformational change of the F protein from prefusion to postfusion poses challenges for vaccine development. In recent years, with the elucidation of the RSV PreF protein structure, researchers have identified at least seven antigenic sites on the surface of F protein.^44^ Although approximately 50% of the surface is shared between the PreF and PostF states, the most sensitive neutralizing sites, site Ø and site V, are only present in the PreF conformation, suggesting that the PreF protein can induce the production of potent neutralizing antibodies.^45^, ^46^ In addition, F protein in the virus surface of the formalin‐inactivated vaccine transitions from the prefusion conformation to the postfusion conformation, which may account for the vaccine's suboptimal efficacy.^47^ This also underscores the importance of a stable PreF protein conformation as a key to antigen development.

The RSV PreF protein represents an ideal target for vaccine development, but the protein is extremely labile and prone to conformational changes. Given the potential for immuno‐focusing offered by the PreF protein antigen, current research directions in RSV vaccine development are concentrated on the design of stable PreF protein antigens and the study of mechanisms to maintain their stability.^19^ Structural biology‐based research has identified various protein engineering strategies for stabilizing the F protein in the prefusion conformation, which are crucial for RSV vaccine development. These strategies include the addition of non‐natural disulfide bonds, hydrophobic filling mutations in cavities, proline mutations, electrostatic interaction mutations, and the addition of heterologous trimerization domains.^48^, ^49^, ^50^, ^51^, ^52^ The first prefusion‐stable subunit candidate vaccine, DS‐Cav1, demonstrated the ability to induce potent neutralizing antibodies in humans during Phase 1 clinical trials.^53^ Building upon these findings, further developed vaccines such as the Arexvy recombinant protein vaccine, Abrysvo recombinant protein vaccine, and mRESVIA mRNA vaccine, all utilize the RSV PreF protein as the immunogen.^29^, ^54^ Their efficacy and safety have been validated, culminating in successful commercialization.

#### 2.1.2 Types of RSV vaccines

**FIGURE 2:**
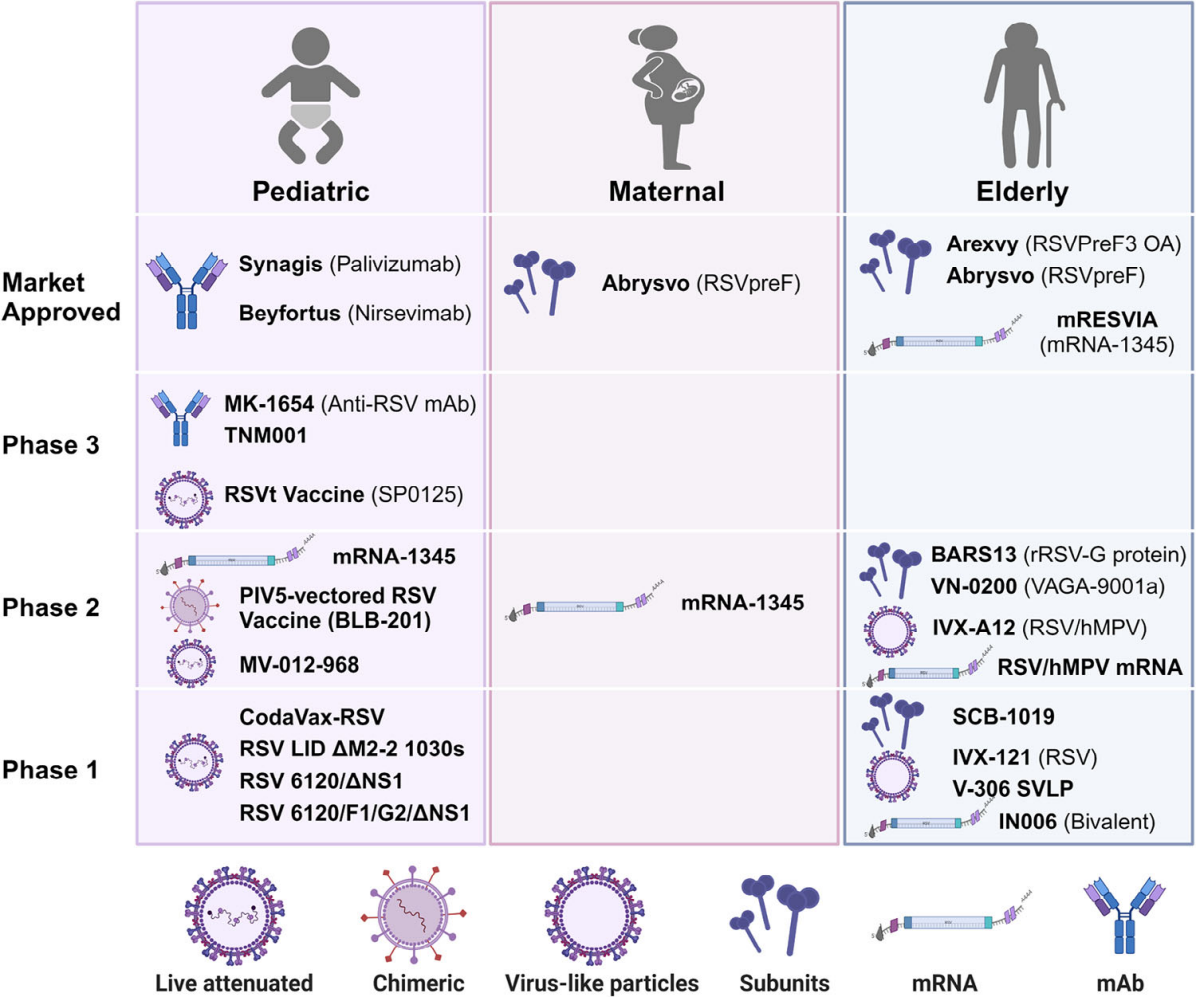
RSV vaccine and mAb candidates. Vaccine and mAb candidates can be categorized into three target populations: (1) pediatric, (2) maternal, and (3) elderly. Vaccine and antibody development phases include the market approval phase, Phase 3, Phase 2, and Phase 1. The different platforms for vaccine and antibody development include (1) live attenuated vaccines, (2) live‐attenuated chimeric vaccines, (3) recombinant virus‐liker particles vaccines, (4) recombinant subunits vaccines, (5) mRNA vaccines, and (6) antibodies. Figure 2 is created using BioRender (https://www.biorender.com/).

In recent years, with the advancement of molecular biological research on RSV and the application of novel vaccine design strategies, the development of RSV vaccines has progressed rapidly. Currently, a multitude of candidate vaccines are under development globally, including inactivated virus vaccines, live attenuated vaccines (LAVs), recombinant protein subunit vaccines, viral vector vaccines, mRNA vaccines, and others^18^ (Figure 2).

In 1966, the formalin‐inactivated whole virus FI‐RSV vaccine led to an exacerbation of disease in vaccinated children upon RSV infection, tragically resulting in the deaths of two children.^55^ The enhanced respiratory/RSV disease (ERD)^56^ caused by the FI‐RSV vaccine prompted a halt in RSV vaccine trials for several decades and indirectly led to stringent safety review requirements for all subsequent RSV candidate vaccines. LAVs of RSV can mimic the infection by a live virus, and attenuation is primarily achieved through traditional methods such as heat or chemical treatment, or by reverse genetic techniques, for example, through mutations or deletions in the RSV M2, NS2/NS1, and L genes, which restrict RSV replication in the vaccinee.^57^, ^58^, ^59^, ^60^ The main challenge with LAVs is balancing safety (attenuation) with the immune response to develop a safe and effective vaccine candidate. Recombinant protein subunit RSV vaccines typically consist of purified viral proteins and adjuvants. Early vaccines based on the PostF conformation were unsuccessful. Current vaccines primarily contain the PreF conformation as the immunogen, such as GSK's Arexvy vaccine and Pfizer's Abrysvo vaccine.^61^ Additionally, several companies are developing nanoparticle vaccines targeting the F protein, such as Novavax's ResVax.^62^ Advaccine's ADV110 vaccine, based on the RSV G protein, demonstrated good safety, tolerability, and immunogenicity in Phase 2 clinical trials.^63^, ^64^ Viral vector vaccines for RSV include Bavarian Nordic's MVA‐BN‐RSV vaccine^65^, ^66^, ^67^ carrying the RSV F, G, N, and M2 proteins, and Janssen's Ad26.RSV.preF vaccine,^68^, ^69^, ^70^, ^71^ designed to express the RSV PreF protein. However, their clinical development has been fraught with challenges, leading to the discontinuation of these programs. RSV mRNA vaccines contain sequences encoding the PreF protein, capable of inducing robust humoral and cellular immune responses. Moderna's mRNA‐1345 vaccine (trade name mRESVIA) has shown good stability and efficacy and has been approved for marketing.^72^, ^73^, ^74^, ^75^

In summary, although the production process of inactivated viral vaccines is mature, fewer studies have been conducted due to the potential ERD effect. In contrast, LAVs, viral vector vaccines, and mRNA vaccines can mimic the natural infection process, simultaneously inducing high titers of neutralizing antibodies and CD8^+^ T cell immune responses, generating Th1‐biased immune responses, and not causing ERD, and therefore are expected to be used for the prevention of RSV infections in infants and young children. In addition, recombinant protein subunit vaccines usually maintain the F protein conformation in the PreF state through targeted mutagenesis, thus inducing strong neutralizing antibodies, and are currently the most effective form of RSV vaccine for pregnant women and the elderly population.

#### 2.1.3 Vaccine development strategies for different populations

The target populations for RSV vaccines encompass newborns (less than 6 months of age), infants and young children (6 months to 5 years), pregnant women, and the elderly.^3^ Given the significant differences in infection risk, immune status, and the potential for complications among these various demographic groups, distinct vaccine development strategies are warranted for each population.^43^

The immune system of newborns aged 0–6 months is not fully developed, leading to the highest susceptibility to severe manifestations of RSV infection among all the age groups. Concurrently, the window for active immunization is narrow, making it challenging to induce timely and effective immune protection. Therefore, passive immunization in neonates can be achieved through the immunization of pregnant women with PreF subunit vaccines. Currently, only Pfizer's bivalent RSV vaccine, Abrysvo, has been approved for use in pregnant women during weeks 32–36 of gestation.^76^ Following vaccination, pregnant women mount an immune response characterized by the production of immunoglobulins G (IgG) and A (IgA), which serve to prevent lower respiratory tract disease (LRTD) and severe LRTD caused by RSV in infants from birth to 6 months.^77^ Specifically, IgG antibodies are transferred in substantial quantities from the maternal circulation to the fetus via the placenta, an endocytic process mediated by the IgG‐specific transport receptor FcRn within the syncytiotrophoblast cells.^78^ This transplacental transfer confers passive immune protection to the fetus through the acquisition of maternal antibodies. Furthermore, IgA antibodies, which constitute 80–90% of the total Igs in breast milk, are transmitted to the newborns through breastfeeding, thereby providing RSV‐specific antibodies to the infant.^79^

Infants and young children (6 months to 5 years of age) are also at significant risk from RSV infections, and to date, there is no safe and effective vaccine available for this age group. An RSV vaccine suitable for this age group must ensure that it does not cause ERD, and the development of such a vaccine presents considerable challenges.^80^ Similar to the FI‐RSV vaccine, recombinant subunit vaccines may induce Th2 or Th17 biased cellular immune responses, which carry the risk of ERD and are not suitable for infants and young children.^81^ In contrast, LAVs, chimeric vaccines, and viral vector vaccines for RSV can mimic natural viral infection through mucosal immunity, inducing neutralizing antibodies and CD8^+^ T cell immune responses, leading to Th1‐biased immune responses that do not cause ERD.^82^ As such, these types of vaccines are the primary focus of research for the prevention of RSV in infants and young children.

Individuals aged 60 years and above who have previously experienced natural RSV infections have developed preexisting immunity, which facilitates a more diversified strategy for vaccine development.^29^

### 2.2 Progress in RSV vaccine research and development

#### 2.2.1 Vaccine progress for preventing RSV infection in older adults

Arexvy (RSVPreF3 OA) vaccine, developed by GSK, was approved in May 2023 and represents the first RSV preventive vaccine globally approved by the United States Food and Drug Administration (US FDA) for the prevention of RSV‐mediated LRTD in individuals over the age of 60 years.^83^ The Arexvy vaccine is composed of an antigen and an adjuvant, with the antigen being lyophilized to ensure stability. The antigen includes 120 µg of recombinant prefusion F glycoprotein antigen (RSV PreF3) derived from the CHO cell line, representing a subtype of RSV‐A viral protein. The adjuvant utilizes the proprietary AS adjuvant system's liposome‐based AS01E, containing 25µg each of QS‐21 (Quil A) and MPL (monophosphoryl lipid A). This formulation promotes the recruitment and activation of antigen‐presenting cells in the draining lymph nodes following intramuscular injection. As a result, it leads to the generation of RSV PreF3‐specific Th1 CD4^+^ T cell immune responses and the induction of a neutralizing antibody response. The market authorization of Arexvy was primarily based on the positive results of the AReSVi‐006 trial (NCT04886596), a randomized, placebo‐controlled, single‐blind, global multicenter Phase 3 clinical study. This trial assessed the efficacy of a single lifetime administration and annual administration of Arexvy in elderly individuals over 60, demonstrating an 82.6% efficacy against RSV‐LRTD, 94.1% against severe RSV‐LRTD, and 71.7% against RSV‐acute respiratory infections (ARIs) within a single epidemic season.^84^ A single dose of Arexvy was further shown to provide 67.2% protection against RSV‐LRTD across two complete RSV seasons.^85^ Furthermore, the administration of a booster prior to the second epidemic season, following the initial dose in the first season, yielded efficacies of 67.1, 78.8, and 60.3% against RSV‐LRTD, severe RSV‐LRTD, and RSV‐ARI, respectively, over two RSV seasons, without hospitalization due to RSV infection among participants.^83^ In summary, a single dose of Arexvy effectively prevents RSV‐related diseases in individuals 60 years and older over two consecutive RSV epidemic seasons, with a favorable safety profile. The booster dose did not confer additional protective efficacy, and further confirmation of the optimal booster vaccination schedule is required. In June 2024, the US FDA expanded the indications for Arexvy to include adults at high risk for RSV between the ages of 50 and 59 years,^31^ based on the positive results of a Phase 3, placebo‐controlled, randomized study (NCT05590403). This study established the noninferiority and safety of the immune response to a single dose of Arexvy in participants aged 50–59 years with increased risk for RSV‐LRTD, compared with the older population over 60 years.^86^ Alarmingly, an imbalance in the rates of Guillain‐Barré Syndrome (GBS) between vaccine and placebo recipients was identified in clinical trials supporting licensure of Arexvy.

Abrysvo (RSVpreF) vaccine, developed by Pfizer Inc., received US FDA approval in May 2023 for the prevention of LRTD caused by RSV in individuals aged 60 years and older.^87^ Abrysvo is a nonadjuvanted, bivalent subunit vaccine, comprising 60 µg each of the prefusion F proteins from RSV A and B subtypes, designed to provide comprehensive protection against both RSV A and B group infections.^88^, ^89^ The market authorization of Abrysvo was primarily based on the positive outcomes of the RENIOR trial (NCT05035212), an ongoing randomized, double‐blind, placebo‐controlled, international Phase 3 clinical trial involving 34,284 participants. The trial results demonstrated that a single dose of the vaccine had a vaccine efficacy (VE) of 66.7% for preventing LRTI with at least two symptoms, 85.7% for preventing those with at least three symptoms, and 62.1% for preventing ARIs caused by RSV. Additionally, the single dose of Abrysvo showed an efficacy of 77.8% against RSV‐associated LRTD with three or more symptoms in a subsequent full RSV season in adults aged 60 years or older.^90^ Similarly, Abrysvo showed a probabilistic event of GBS, which deserves attention and observation in further clinical studies.

mRESVIA (mRNA‐1345) vaccine, developed by Moderna, Inc., was granted US FDA approval in May 2024 for the protection of adults aged 60 years and older from RSV‐LRTD. As the third RSV vaccine globally and the first RSV mRNA vaccine worldwide, mRESVIA utilizes lipid nanoparticles (LNP) delivery technology, containing 50µg of mRNA‐1345 encoding the stabilized RSV prefusion F glycoprotein.^72^ The F glycoprotein has undergone structural modification and codon optimization to augment the stability and potency of the mRNA vaccine.^73^ The market introduction of mRESVIA was primarily based on positive data from the ConquerRSV trial (NCT05127434), a randomized, double‐blind, placebo‐controlled global Phase 2–3 clinical study, enrolling approximately 35,000 adults aged 60 years and older. With a median follow‐up of 3.7 months, the vaccine demonstrated an efficacy of 83.7% against RSV‐LRTD with two or more symptoms and 82.4% against RSV‐LRTD with three or more symptoms.^74^ Unexpectedly, the long‐term protective efficacy of the mRESVIA vaccine showed a significant decline, with an efficacy of 50.3% against RSV‐LRTD with two or more symptoms and 49.9% against more severe infections with at least three symptoms after 18 months. Additionally, the mRNA RSV candidate vaccine SYS6016 by CSPC Pharmaceutical Group is undergoing clinical trials in China; and the bivalent RSV mRNA vaccine developed by InnoRNA has received clinical trial approval from the US FDA and is pending clinical application in China.

#### 2.2.2 Progress of maternal vaccines for preventing infant RSV infection

Abrysvo (RSVpreF) vaccine, beyond its application for elderly individuals aged 60 years and older, received regulatory approval in August 2023 for administration to pregnant women during the gestational period from the 32nd to the 36th week.^91^, ^92^ This approval is intended to elicit active immunity, thereby preventing LRTD and severe LRTD in infants from birth to 6 months of age caused by RSV.^93^ The rationale for extending the target demographic of the Abrysvo vaccine to include pregnant women is anchored in the positive outcomes of the MATISSE trial (NCT04424316). At postpartum intervals of 90 and 180 days, the efficacy of Pfizer's RSV vaccine in mitigating medically attended RSV‐related severe LRTD was reported to be 81.8 and 69.4%, respectively.^76^ This evidence supports the effectiveness of RSVpreF vaccination during pregnancy in preventing RSV‐associated LRTD in infants, with no significant safety concerns observed in vaccinated pregnant women or their infants. However, a preterm birth rate of 5.7% for infants in the Abrysvo vaccine group and 4.7% in the placebo group was observed in clinical trials. Although the difference was not significant, an association between the vaccine and preterm labor cannot be ruled out.

#### 2.2.3 Progress of vaccine preventing pediatric RSV infection

Currently, within the pediatric population, particularly infants aged 6 months to children up to 5 years, numerous clinical trials for RSV attenuated live vaccines and viral vector vaccines have been initiated. These vaccines are capable of being administered via the intranasal route, which can induce a triple‐tiered immune response: systemic immunity, mucosal immunity comprising secretory IgA and mucosal tissue‐resident memory T cells, as well as trained innate immunity.^94^ The intranasal vaccination approach not only mitigates disease severity but also prevents viral colonization in the mucosal epithelium of the upper and lower respiratory tract, resists early infection, and interrupts viral infection and transmission pathways. However, clinical trials for viral vector vaccines have encountered significant challenges, with several programs being discontinued. The attenuated live vaccines under development predominantly utilize reverse genetics techniques for the virus. These involve the deletion of genes encoding the RSV NS2 and M2‐2 proteins, codon deoptimization, and site‐directed mutations and modifications of the RSV L protein, thereby generating vaccine strains with attenuated virulence compared with the wild‐type strains.^58^, ^95^

RSVt (SP0125) is a temperature‐sensitive attenuated strain codeveloped by Sanofi and the National Institute of Allergy and Infectious Diseases (NIAID). It has recently received approval for a global Phase 3 clinical trial (NCT06252285) to assess the efficacy, immunogenicity, and safety of the RSVt vaccine in infants aged between 6 and 22 months. The RSVt vaccine is an attenuated strain of RSV ΔNS2/Δ1313/I1314L, which has undergone genetic modifications including the deletion of the NS2 gene, the deletion of the 1313th amino acid in the L gene, and a mutation at the 1314th amino acid of the L gene.^96^ In Phase 1 clinical trials (NCT03227029 and NCT03422237), the vaccine yielded positive outcomes, demonstrating significant therapeutic efficacy and the ability to induce a broad and long‐lasting immune response. Two doses of the RSVt vaccine were able to elicit an antibody response in 93% of the subjects enrolled in the study.^60^

MV‐012‐968 is a vaccine candidate developed by Meissa Vaccines, primarily targeting the codon deoptimization of genes encoding the RSV G, NS1, and NS2 proteins, while also deleting the gene encoding the SH protein and eliminating the secreted G protein, thereby achieving the goal of attenuation. A Phase 1 trial conducted among children aged 6–36 months (NCT04909021) confirmed the safety of the vaccine and indicated that the neutralizing antibodies were boosted in 78% of the study population.

CodaVax‐RSV is a candidate intranasal attenuated live vaccine developed by Codagenix Inc. The vaccine employs a gene‐editing approach that introduces hundreds of point mutations across the genome, including codon deoptimization of the L protein‐encoding gene and amino acid mutations in the N, P, M2‐1, and L proteins, which collectively prevent reversion to a wild‐type phenotype.^97^ This attenuated vaccine demonstrates favorable safety profiles and is capable of eliciting both systemic and mucosal immune responses.^98^

### 2.3 Unresolved issues and remaining challenges of RSV vaccine

The year 2023 has been hailed as the “first year of RSV vaccine commercialization.” After more than six decades of efforts, the Arexvy recombinant protein vaccine, Abrysvo recombinant protein vaccine, and mRESVIA mRNA vaccine have successively entered the market and have been approved for the prevention of RSV‐associated LRTD in individuals aged 60 years and above, as well as for administration to pregnant women from 32 to 36 weeks of gestation to prevent RSV‐associated LRTD in infants from birth to 6 months. However, the quest for RSV prevention is far from complete, with numerous unresolved challenges remaining: (1) the absence of vaccines for children over 6 months old, (2) ongoing safety concerns of the vaccines, (3) the duration of protection offered by the vaccines, (4) the role and necessity of adjuvants in the development of RSV vaccine, (5) a variety of issues requiring resolution throughout the vaccine development process, and (6) the feasibility and efficacy of developing multivalent and multicomponent vaccines.

#### 2.3.1 Absence of vaccines for children older than 6 months

Currently, the primary challenge in RSV vaccine development is the urgent need to provide safe and effective vaccines for seronegative infants and young children aged 6 months and older. However, due to the immature immune systems of infants and the incomplete understanding of the ERD mechanism,^56^, ^99^ there is no definitive method to avoid the risk of ERD,^100^ making the development of vaccines for children over 6 months, especially seronegative children, a formidable task. Fortunately, data from several clinical trials indicate that RSV LAVs are among the most competitive candidates for seronegative infants and are expected to represent a new breakthrough following the RSV structural vaccines.^58^, ^101^, ^102^ However, achieving a reasonable balance between the safety, immunogenicity, and genetic stability of RSV LAVs is extremely difficult and is key to advancing RSV LAVs vaccine research. At present, with the development of reverse genetics technology, gene editing technology, and synthetic biology, significant progress has been made in balancing the attenuation and immunogenicity of RSV LAVs.^59^, ^103^ Most importantly, the RSVt vaccine jointly developed by Sanofi and NIAID entered Phase 3 clinical trials in February 2024, planning to enroll 6300 infants aged 6–22 months to assess the efficacy, immunogenicity, and safety of the intranasally administered RSV vaccine for infants (RSVt) compared with placebo. Therefore, it is reasonable to believe that in the near future, RSV vaccines for children aged 6 months and older will be commercialized.

#### 2.3.2 Ongoing safety concerns of RSV vaccine

The safety of RSV vaccines is of paramount importance, primarily due to the unique characteristics of the target populations, such as seronegative children and pregnant women. Furthermore, the risk of GBS following RSV vaccination in the elderly population aged 65 years and older remains to be thoroughly assessed.^104^, ^105^

First, the FI‐RSV vaccine exacerbated the severity of RSV disease in seronegative children in clinical settings, leading to the two tragic pediatric deaths and subsequently causing a prolonged halt in the development of RSV vaccines for children. After more than six decades of research, it is preliminarily considered that a contributing factor to ERD may involve the production of a substantial amount of virus‐specific non‐neutralizing antibodies following immunization with inactivated vaccines, whereas neutralizing and fusion‐inhibiting antibodies are relatively weak. Furthermore, the presence of significant immune complex deposition in the lungs, along with a substantial increase in eosinophils and IgE production, has directed the failure of FI‐RSV toward a Th2‐biased immune response. However, the precise mechanisms underlying the ERD phenomenon remain unclear. The development of RSV vaccines should focus on evaluating the strength of neutralizing antibodies induced by immunogens and the balance of Th1/Th2 immune responses. Therefore, preventing the occurrence of ERD, ensuring the safety of the vaccine, remains a critical aspect of pediatric vaccine development.^106^

Subsequently, the safety assessment of RSV vaccines for pregnant women is of critical importance. The Phase 3 clinical trial (NCT04605159) of the RSVPreF3‐Mat vaccine, developed by GSK, demonstrated that the vaccine's efficacy in preventing any RSV‐related LRTD was 65.5%, and the efficacy in preventing severe RSV‐related LRTD was 69.0%. However, the clinical trial was prematurely terminated due to a significantly higher preterm birth rate in the vaccine group compared with the placebo group, which was 6.8 and 4.9%, respectively.^107^ Additionally, there was a slight increase in neonatal mortality in the vaccine group, although not statistically significant. It is hypothesized that the higher preterm birth rate in the vaccine group compared with the placebo group may ultimately lead to the discontinuation of the RSVPreF3‐Mat vaccine development. It remains unclear if the safety concerns in the RSVPreF3‐Mat trial represent genuine risks or are coincidental occurrences. Researchers have conducted multiple post hoc analyses, but the mechanism by which RSVPreF3‐Mat vaccination may increase the risk of preterm birth has yet to be determined.^108^ Furthermore, the US FDA‐approved Abrysvo vaccine showed a preterm birth rate of 5.7% in the vaccine group and 4.7% in the placebo group.^76^ Although the difference is not significant, it does not rule out a potential association between vaccination and preterm birth. Consequently, there is an urgent need for postmarketing surveillance to assess the association between the use of Abrysvo vaccine and the risk of preterm birth, weighing the benefits and potential risks to both the mother and the fetus during pregnancy.^109^

Postmarketing surveillance of the Arexvy and Abrysvo vaccines has identified an imbalance in the incidence of GBS between recipients of the vaccine and placebo. The US FDA is conducting a postlicensure safety study on RSV vaccines, employing two methodological approaches: observed versus expected analysis, and the self‐controlled case series analysis. The results from two different types of analyses of potential GBS risk following RSV vaccination are mixed and highly uncertain. These analyses do not provide clear, conclusive evidence of an elevated risk of GBS and an elevated risk cannot be ruled out at this time. US FDA is conducting medical chart review on GBS cases and will continue to evaluate the safety of RSV vaccines as more data are available. Although the risk associated with GBS following vaccination remains uncertain, the US FDA maintains that the benefits of RSV vaccination outweigh the potential risks. However, the US Centers for Disease Control and Prevention (CDC) and the Advisory Committee on Immunization Practices (ACIP) have revised their vaccination recommendations for the elderly population. Contrary to previous endorsements for individuals aged 60–74 years, the current guidance no longer advises vaccination for those without identified RSV risk factors within this age bracket.

#### 2.3.3 The duration of protection offered by RSV vaccines

The RSV vaccine was just approved for market entry in 2023, and the primary endpoints supporting the safety and efficacy data of the vaccine's BLA product only evaluated the protectiveness of a single RSV season, all of which were interim analyses (less than 1 year), thus the duration of immune protection induced by the vaccine in the real world remains uncertain. Moreover, multiple studies have shown that even if the vaccine can induce highly protective antibodies, the decline in viral titer over time leads to a decrease in VE. Currently, GSK, Pfizer, and Moderna have each published the efficacy results of their vaccines over two RSV seasons. Among them, GSK's single‐dose Arexvy achieved an overall protective efficacy of 74.5% during a median follow‐up period of 18 months (Season 1+2). However, looking solely at Season 2, efficacy of the Arexvy vaccine dropped to 56.1%.^85^ Pfizer's single‐dose Abrysvo, during a median follow‐up period of 13.9 months, achieved an overall protective efficacy of 78.6% against three or more RSV‐LRTDs, yet the efficacy against two or more RSV‐LRTDs dropped to only 48.9%. Moderna's single‐dose mRESVIA, during a median follow‐up period of 18 months, showed the vaccine's efficacy in preventing RSV‐related LRTD with two or more symptoms to be 50.3%, and the efficacy in preventing more severe infections with at least three symptoms to be 49.9%. In summary, as time progresses, the efficacy of RSV vaccines appears to decline, suggesting that periodic booster vaccinations may be necessary.^110^

#### 2.3.4 Role and necessity of adjuvants in RSV vaccine development

Adjuvants are nonspecific immune enhancers, which can enhance the body's immune response to antigens or modulate the type of immune response by assisting in antigen presentation. Three types of adjuvants are commonly used: immunomodulatory molecule‐based adjuvants, antigen‐delivery adjuvants, and combination adjuvants. Only seven adjuvants have been approved by the US FDA for market use, including aluminum adjuvant, MF59, AS03, AS01, AS04, CpG 1018, and matrix‐M adjuvant. The safety of RSV vaccines is dependent on the balance of Th1/Th2 immune responses, necessitating the induction of strong neutralizing antibodies and RSV‐specific CD8^+^ T‐cell responses, while avoiding the production of non‐neutralizing antibodies, the induction of Th2‐biased CD4^+^ T‐cell responses, and the production of IL‐4, IL‐5, IL‐13, and so on. Therefore, the choice of adjuvant for RSV vaccines is crucial.^111^ Significant differences between the GSK Arexvy vaccine and the Pfizer Abrysvo vaccine lie in the fact that the Arexvy vaccine utilizes the AS01E adjuvant, whereas the Abrysvo vaccine does not contain an adjuvant. Researchers have found that the AS01E adjuvant used in the GSK Arexvy vaccine can significantly enhance Th1 immune response, inducing a better immune response in the elderly population, thus achieving a balance of Th1/Th2 immunity.^112^ Pfizer, on the other hand, conducted comparative analyses in clinical trials to assess the impact of various adjuvants, including aluminum hydroxide and CpG, against the absence of an adjuvant, and the results showed no significant increase in neutralizing antibody titers with the addition of adjuvants.^88^, ^113^ Therefore, the Pfizer Abrysvo vaccine was ultimately formulated without an adjuvant. The side effects of not adding an adjuvant are greatly reduced, which may be one of the reasons why the Pfizer Abrysvo vaccine can be administered to pregnant women. In summary, the use of adjuvants in the development of RSV vaccines is still inconclusive, and whether adjuvants with different mechanisms of action are suitable for different types of RSV vaccines remains to be further studied.

#### 2.3.5 Various issues to be resolved in vaccine development process

RSV vaccines confront a variety of challenges across the early development, preclinical research, and clinical trial phases. There are several pressing issues that currently demand solutions, including limitations in the animal models used for immunological evaluation, the lack of clarity regarding the surrogate endpoints for vaccine immunogenicity, and the application of human challenge studies.

RSV animal models play a pivotal role in investigating the mechanisms of RSV infection, as well as in the evaluation of vaccines and therapeutic strategies. Preclinical evaluation of RSV vaccines in animal models primarily encompasses the assessment of vaccine immunogenicity, detection of adverse reactions and toxicity, and the evaluation of in vivo protective efficacy. Commonly utilized RSV animal models include nonhuman primates, mice, cotton rats, guinea pigs, cattle, and sheep.^114^ However, except for chimpanzees, other animal models exhibit only semi‐permissive characteristics for RSV and are unable to fully replicate the immune responses and pathogenic conditions of RSV in humans, posing significant challenges in evaluating the efficacy of vaccines. Notably, the susceptibility of cotton rats to RSV infection is more than 100 times greater than that of mice, and they exhibit the ERD phenomenon. After intranasal inoculation with RSV, both the upper and lower respiratory tracts of cotton rats are susceptible to infection, and significant pathological changes occur in the respiratory system postinfection. Consequently, cotton rats are considered a suitable model for studying RSV vaccine immunology, particularly as the preferred animal for evaluating vaccine safety in humans.^115^ However, cotton rats are not a conventional animal model and are relatively difficult to obtain. Furthermore, research has shown that several candidate vaccines exhibit complete protective efficacy in rodent models but perform poorly in chimpanzees and other NHPs, such as African green monkeys. To date, no RSV animal model that can fully replicate the severity of the human disease and is easily accessible has been identified.

The main serological indicators for the assessment of immunogenicity as surrogate endpoints include neutralizing antibodies, binding antibodies, bactericidal antibodies, and hemagglutination‐inhibiting antibodies. Currently, surrogate endpoints for the immunogenicity of many vaccines have been proposed and widely accepted, such as those for influenza virus vaccines, poliovirus vaccines, rabies virus vaccines, hepatitis B virus vaccines, measles virus vaccines, and varicella‐zoster virus vaccines.^116^, ^117^ The key to RSV vaccine development lies in evaluating the strength of neutralizing antibodies induced by the immunogen and the balance of Th1/Th2 immune responses. Neutralizing antibodies and RSV‐specific CD8^+^ T cell responses are important indicators for assessing the efficacy of RSV vaccines. However, the impact of binding antibodies, CD4^+^ T cell responses, and mucosal immunity on VE remains unclear. Currently, there is a dearth of immunological surrogate endpoints and standardized serological markers correlated with protective immunity in RSV vaccine clinical trials. This absence hampers the development of straightforward tools to measure the protective efficacy post‐RSV vaccination. Consequently, there is an urgent need to establish and standardize methods for high‐throughput analysis of RSV neutralizing antibody titers, and to determine scientifically sound and reasonable immunological surrogate endpoints and serological markers for RSV vaccine clinical trials.

At present, there is a multitude of RSV vaccine candidates in the global research pipeline, with many having entered the clinical trial phase. However, for RSV vaccines, large‐scale efficacy trials can only be conducted during the epidemic season and within endemic regions, and various factors may lead to the failure or delay of vaccine clinical trials. Currently, human challenge trials are being increasingly utilized to rapidly screen candidate vaccines, ensuring that the most promising candidates proceed to clinical sites and support regulatory decision‐making.^118^ Human challenge trials have been employed in the development of RSV vaccines (NCT04690335), allowing for preliminary efficacy and safety data to be obtained with fewer subjects vaccinated, which is faster compared with traditional clinical trials.^65^, ^89^ Moreover, RSV human challenge trials can enhance the understanding of the pathogenesis of the disease and the correlation between vaccine‐induced immune responses and protection, providing more reference information for vaccine design. However, human challenge trials involve the intentional exposure of healthy volunteers to the RSV virus, which carries inherent risks and uncertainties.^119^ Consequently, these trials necessitate the presence of robust clinical facilities, a well‐structured recruitment plan, and vigilant monitoring and follow‐up procedures to ensure the safety and well‐being of the participants. In summary, human challenge trials for RSV vaccines can accelerate the pace of vaccine development and better determine the safety and immunogenicity of the vaccines, but the scientific soundness, rational, and feasibility of the trials should be fully assessed and demonstrated to better balance the risks and benefits of the trials.

#### 2.3.6 The feasibility and efficacy of multivalent and multicomponent vaccines

The RSV virus is characterized by two subtypes, A and B, which exhibit certain antigenic differences. Additionally, multiple viral variant strains circulate during different epidemic seasons. Although the RSV F protein is considered to have broad‐spectrum properties, the vaccine's protective efficacy against different subtypes requires further investigation.^120^ The GSK Arexvy vaccine and the Pfizer Abrysvo vaccine also exhibit a notable difference in their composition; the Arexvy vaccine utilizes antigens specific to the A subtype, whereas the Abrysvo vaccine contains antigens from both the A and B subtypes. For the Arexvy vaccine, a significant decline in protective efficacy against RSV‐B was observed over a median follow‐up period of 18 months, which consequently reduced the overall protective efficacy of the vaccine. In summary, the comparative efficacy of monovalent versus multivalent vaccines remains to be further explored. However, preliminary data suggest that a multivalent RSV vaccine encompassing both the A and B subtypes may be necessary to ensure comprehensive protection.

Furthermore, respiratory viruses often exhibit seasonal patterns of prevalence. The development of combination vaccines has the potential to significantly reduce the number of injections required, achieving the effect of “one shot for multiple protections.” Currently, there is a robust pipeline of research into combination vaccines for respiratory viruses, which primarily includes types such as multivalent vaccines, concurrent vaccinations, and viral vector‐based chimeric vaccines (Table 1). However, the feasibility and efficacy of these multivalent and multicomponent vaccines still await confirmation through clinical trial results.

**TABLE 1:** The clinical trials of RSV multivalent and multicomponent candidate vaccines.

Vaccine types	Vaccine	Pathogens targeted	Platform	Sponsor	Phases	NCT number
Combination vaccines	RSV/hMPV vaccine candidate	RSV+hMPV	unknown	Sanofi Pasteur	Phase 1|2	NCT06134648
	RSV/hMPV mRNA LNP	RSV+hMPV	mRNA	Sanofi Pasteur	Phase 1	NCT06237296
	IVX‐A12 (IVX‐121+IVX‐241)	RSV+hMPV	VLP	Icosavax, Inc.	Phase 2	NCT05903183
	IVX‐A12 (IVX‐121+IVX‐241)	RSV+hMPV	VLP	Icosavax, Inc.	Phase 1	NCT05664334
	mRNA‐1365	RSV+hMPV	mRNA	ModernaTX, Inc.	Phase 1	NCT05743881
	mRNA‐1045	RSV+influenza	mRNA	ModernaTX, Inc.	Phase 1	NCT05585632
	mRNA‐1230	RSV+influenza+SARS‐CoV‐2	mRNA	ModernaTX, Inc.	Phase 1	NCT05585632
Combined use of vaccines	RSVPreF3 OA and FLU‐QIV	RSV+influenza	Protein subunit+inactivated	GlaxoSmithKline	Phase 3	NCT04841577
	RSVPreF3 OA and FLU‐QIV HD	RSV+influenza	Protein subunit+inactivated	GlaxoSmithKline	Phase 3	NCT05559476
	RSVPreF3 OA and FLU‐aQIV	RSV+influenza	Protein subunit+inactivated	GlaxoSmithKline	Phase 3	NCT05568797
	RSVPreF3 and FLU‐QIV	RSV+influenza	Protein subunit+inactivated	GlaxoSmithKline	Phase 3	NCT05045144
	RSVpreF and modRNA qIRV	RSV+influenza	Protein subunit+mRNA	Pfizer	Phase 1	NCT05788237
	RSV‐F vaccine and seasonal TIV	RSV+influenza	Protein nanoparticle+inactivated	Novavax	Phase 1	NCT01709019
	RSVPreF3 OA and COVID‐19 mRNA vaccine	RSV+SARS‐CoV‐2	Protein subunit+mRNA	GlaxoSmithKline	Phase 3	NCT06374394
	RSVpreF and BNTb162b2	RSV+SARS‐CoV‐2	Protein subunit+mRNA	Pfizer	Phase 2	NCT05886777
Chimeric vaccine	MEDI‐534	PIV3‐vectored RSV vaccine	Viral‐vector‐based vaccines	MedImmune LLC	Phase 1	NCT00493285
	BLB‐201	PIV5‐vectored RSV vaccine	Viral‐vector‐based vaccines	Blue Lake Biotechnology Inc.	Phase 1	NCT05281263
	BLB‐201	PIV5‐vectored RSV vaccine	Viral‐vector‐based vaccines	Blue Lake Biotechnology Inc.	Phase 1|2	NCT05655182
	RSV/Flu‐01E	Flu‐vectored RSV vaccine	Viral‐vector‐based vaccines	Tatyana Zubkova	Phase 1	NCT05970744

Table 1 provides publicly information on clinical trials of RSV multivalent and multicomponent candidate vaccines. Sources of information for these trials are the website https://clinicaltrials.gov/, and investigators, sponsors, funders of vaccine trials.

Abbreviations: RSV, respiratory syncytial virus; hMPV, human metapneumovirus; PIV, parainfluenza virus; QIV, quadrivalent influenza vaccine; VLP, virus‐like particle.

Multicomponent vaccines target not only RSV and human metapneumovirus (hMPV) but also combine protection against influenza viruses and SARS‐CoV‐2. Notably, Sanofi's RSV/hMPV mRNA LNP vaccine represents a bicomponent mRNA‐based vaccine candidate for RSV and hMPV, which is currently undergoing Phase 1 clinical evaluation. The IVX‐A12 vaccine, a collaborative effort between Icosavax and AstraZeneca, is a composite protein virus‐like particle (VLP) vaccine that comprises IVX‐121 (Icosavax's candidate VLP for RSV's prefusion F protein) and IVX‐241 (Icosavax's candidate VLP for hMPV's prefusion F protein). This vaccine is in Phase 2 clinical trials for the prophylaxis of RSV and hMPV infections. ModernaTX's mRNA‐1365 serves as a bicomponent vaccine for RSV and hMPV; mRNA‐1045, which includes mRNA‐1345 (RSV) and mRNA‐1010 (influenza), is a bicomponent vaccine candidate for RSV and influenza; mRNA‐1230, containing mRNA‐1345 (RSV), mRNA‐1010 (influenza), and mRNA‐1273.214 (SARS‐CoV‐2), is a tri‐component vaccine candidate for RSV, influenza, and SARS‐CoV‐2. All these candidates are currently in the Phase 1 clinical trial stage.^121^

Vaccine coadministration is the practice of using two or more vaccines for different pathogens at the same time. It aims to evaluate safety and potential immunological interactions when different vaccines are administered concurrently, as exemplified in studies conducted by GSK and Pfizer, where an RSV vaccine is coadministered with either an influenza or a COVID‐19 vaccine.^122^, ^123^, ^124^ Currently, several Phase 3 clinical trials are underway, and the results are pending further validation.

Viral vector vaccines represent a significant research avenue in the development of RSV vaccines. However, RSV vaccines from companies such as GSK, Vaxart, Johnson & Johnson, and Bavarian Nordic have encountered setbacks when utilizing viral vector technology. The specific reasons for the clinical trial failures are not yet clear, and the current outcomes do not conclusively indicate that the development path of viral vector‐based RSV vaccines is infeasible. The vaccines employed viral vectors such as adenoviruses and poxviruses. Recently, researchers have initiated multiple RSV vaccine development projects based on parainfluenza virus (PIV) and influenza virus vectors. Blue Lake Biotechnology, Inc. has developed a candidate RSV vaccine known as BLB201, which utilizes the PIV5 vector carrying the gene encoding the RSV F protein.^125^ The vaccine is currently in a Phase 1/2a clinical trial, evaluating its safety, tolerability, and immunogenicity in children aged between 8 and 59 months.

## 3 RSV MONOCLONAL ANTIBODY

### 3.1 Foundation, consensus, and resolved issues of RSV monoclonal antibody

Currently, several monoclonal antibody (mAb) products targeting RSV have been commercialized for the passive immunoprophylaxis of RSV infection in infants and young children.^18^, ^54^ The successful introduction of these RSV‐specific mAb products is also contingent upon advancements in structural biology, particularly the elucidation of the antigenic sites of the RSV F protein and the corresponding binding sites of the mAb.

#### 3.1.1 Antibody passive immunization for infectious disease prevention

Passive immunity refers to the acquisition of specific immune capacity by the organism through the passive reception of antibodies, sensitized lymphocytes, or their products, which is used to combat viral and bacterial infections. As early as 1995, the US FDA approved the product RespiGam for the prevention of RSV in high‐risk infants under 2 years of age, such as those born prematurely or with underlying health issues such as bronchopulmonary dysplasia, congenital heart disease, and so on.^126^ RespiGam is a high‐titer RSV intravenous Ig (RSV‐IVIG), which is a polyclonal antibody preparation isolated from the serum of donors with high levels of anti‐RSV neutralizing antibodies. The main component is IgG, but it also contains trace amounts of IgA, IgM, and other serum proteins. RespiGam can directly neutralize RSV, specifically blocking the virus's entry into cells, thereby improving clinical symptoms.^127^ However, due to the inconvenience in the preparation and use of RSV‐IVIG, its application has been limited and its use has gradually decreased.^128^ In summary, passive immunization with RSV‐IVIG and mAb products can be used for the prevention of RSV infection and has a history of use for more than 20 years. With the rapid development of mAb technology, passive immunization for the prevention of RSV infection now primarily utilizes mAbs to ensure the safety and efficacy of the products.

#### 3.1.2 Antigen site analysis of RSV fusion protein

**FIGURE 3:**
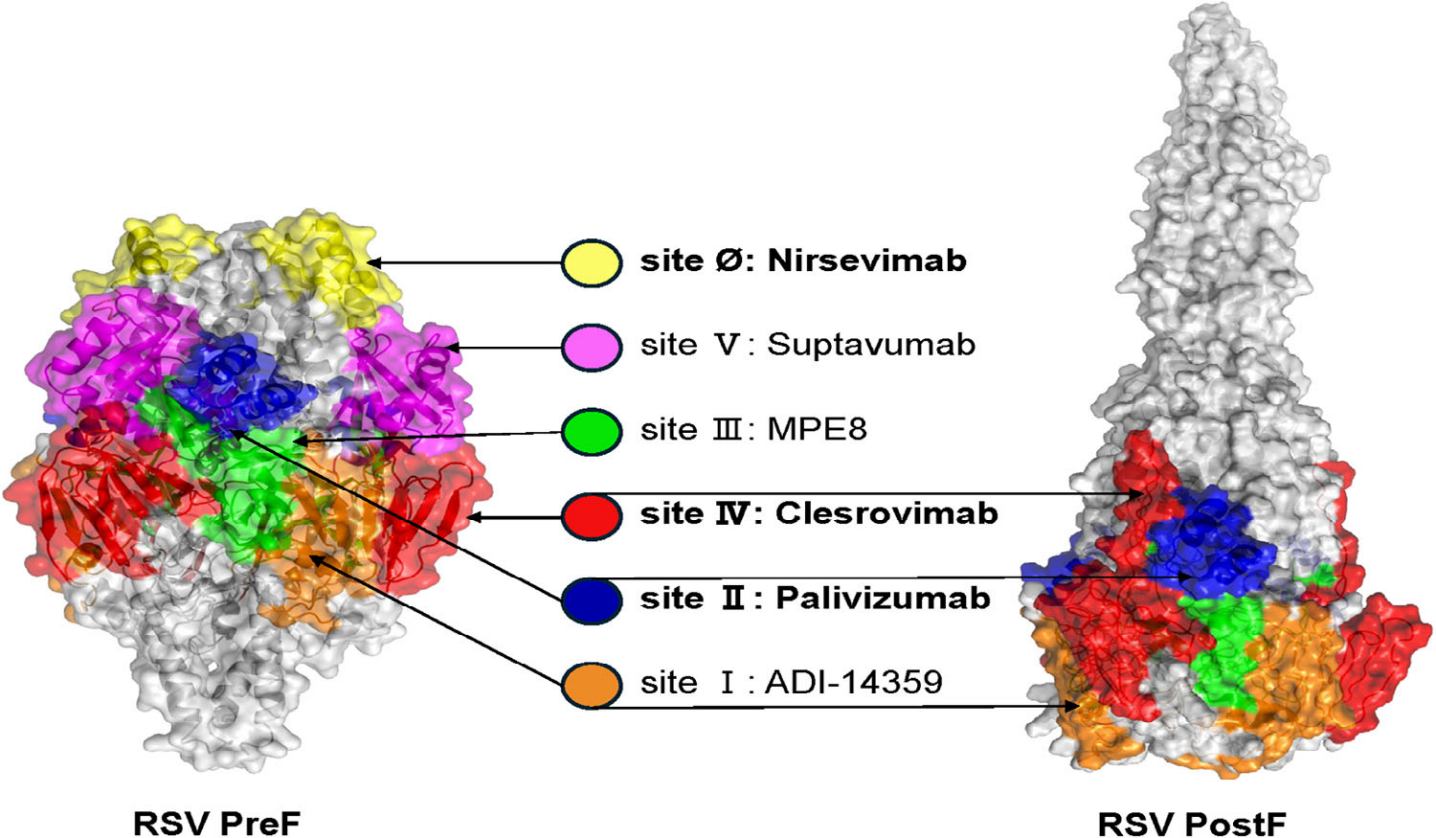
RSV F sites and their respective mAbs. RSV prefusion protein (globular) and postfusion protein (elongated) conformations. RSV PreF PDB: 7LUC; RSV PostF PDB: 3RRT. Antigenic sites are colored for each structure. Site Ø is colored yellow; site V is colored purple; site III is colored green; site IV is colored red; site II is colored dark blue; site I is colored orange. Respective mAbs for each site are shown.

In recent years, with the elucidation of the structure of the RSV F protein and its PreF conformation, researchers have identified at least seven completely distinct antigenic sites on the F protein's surface.^129^, ^130^ They have successfully screened antibodies targeting these different sites, including the 131–2A^131^ mAb for site I, motavizumab,^132^ palivizumab^133^ mAbs for site II, MPE8^134^ mAb for site III, 101F^135^, mAb19^136^ mAbs for site IV, MK‐1654^137^ for site V, and D25^14^, 5C4,^138^ AM22^14^ mAbs for site Ø. Among these, sites I, II, III, and IV are presented in both the PreF and PostF conformations of the protein, while sites V and Ø are exclusively presented in the PreF form. Currently, antibodies against F protein can be classified according to their neutralizing potency from high to low: PreF‐specific site Ø, PreF‐specific site V, site III with PreF > PostF, site IV with PreF and PostF, site II with PreF and PostF, and site I with PostF > PreF (Figure 3). Specifically, site Ø is located at the top of the PreF protein and is formed by the heptad repeat A (HRA) region folded into the globular head. Compared with other antigenic sites, neutralizing antibodies induced by site Ø are potent but exhibit higher variability among different RSV subtypes.

### 3.2 Progress in RSV mAb research and development

The World Health Organization (WHO) has delineated in its document the “WHO preferred product characteristics of mAbs for passive immunization against RSV‐associated disease” that an ideal RSV mAb product should be a high‐quality, safe, and efficacious passive prophylactic agent, with priority given to public health needs. MAb products should be utilized for the prevention of severe RSV disease and mortality in infants under 12 months of age, as well as for reducing the incidence of disease in children under 5 years. Additionally, mAb products must be affordable and accessible, with the capability to be deployed globally, including in low‐ and middle‐income countries (LMICs).^139^ The following will provide a detailed summary of the characteristics of major RSV mAbs, including palivizumab, nirsevimab, and clesrovimab.

#### 3.2.1 Palivizumab (Synagis)

Palivizumab, a humanized mAb of murine derivation developed by MedImmune in conjunction with Astra Zeneca, targets the site II of the F protein, exhibiting moderate neutralizing activity. Identified through hybridoma screening, this mAb has undergone humanization processes, yet retains approximately 5% of its murine origin antibody sequence.^140^ In 1998, it received approval from the US FDA for the prevention of severe LRTI caused by RSV in specific high‐risk pediatric populations.^141^ The indicated population comprises infants born at 35 weeks of gestation or earlier, children with chronic lung disease, and children with congenital heart disease who exhibit significant hemodynamic abnormalities. Palivizumab has a relatively short half‐life of approximately 28 days, necessitating monthly intramuscular injections at a dosage of 15 mg/kg throughout the RSV season, typically commencing in November and lasting for 5 months.^37^ The benefits of palivizumab have been a subject of debate,^142^ with an abundance of research on its efficacy and safety in recent years.^36^ The American Academy of Pediatrics, summarizing extensive clinical data, stated in 2023 that palivizumab should be applied for the prophylaxis of RSV in high‐risk infants and young children at increased risk of hospitalization, such as those with chronic lung disease, congenital heart disease, Down syndrome, immunodeficiency, pulmonary anatomical abnormalities, neuromuscular disorders, and cystic fibrosis, and is not recommended for the prevention of hospitalization in other healthy preterm and term infants.^143^ In conclusion, palivizumab is indicated for a more narrowly defined population and is not suitable for application across all pediatric groups. Moreover, the high production costs and large dosage requirements of palivizumab contribute to a less than optimal market share.

#### 3.2.2 Nirsevimab (MEDI8897, Beyfortus)

Nirsevimab, a recombinant human IgG1κ mAb developed by Astra Zeneca in partnership with Sanofi, is a fully human antibody derived through B‐cell sorting that targets the site Ø of the F protein, demonstrating over 50‐fold greater neutralizing activity compared with palivizumab. Utilizing the M252Y/S254T/T256E (YTE) mutational technique, nirsevimab enhances its affinity for the neonatal Fc receptor at lower pH, thereby evading lysosomal degradation and protracting its half‐life. The mean half‐life of nirsevimab, ranging from 85 to 117 days, is approximately tripartite that of palivizumab, offering protection throughout the RSV season with a single dose.^144^ In November 2022, nirsevimab gained regulatory approval in the European Union and the United Kingdom for the prophylaxis of RSV‐induced LRTD in the inaugural RSV season of neonates and infants.^39^ Subsequently, in July 2023, the US FDA accorded its approval, with the ACIP of the US CDC recommending its use in infants less than 8 months of age born or entering the RSV season, as well as in infants aged 8–19 months with an elevated risk of severe RSV disease during their second RSV season.^40^ The efficacy and safety profiles of nirsevimab are substantiated by two pivotal global multicenter clinical trials (NCT02878330 and NCT03979313), which assessed its efficacy, safety, and pharmacokinetics (PK) by monitoring healthy term and preterm infants entering their first RSV season.^145^, ^146^, ^147^ The Phase 3 clinical study enrolled neonates with a gestational age exceeding 35 weeks, while the Phase 2b trial included neonates with a gestational age between 29 and 35 weeks. Clinical trial outcomes revealed that nirsevimab's protective efficacy against RSV‐associated medical visits for LRTI was 79.0%, hospitalization due to RSV‐associated LRTI was 80.6%, and ICU admissions due to RSV‐associated LRTI was 90.0%.^148^ Real‐world studies from countries such as France, Germany, Spain, and the United States report that nirsevimab's protective efficacy against RSV‐related hospitalizations is between 85% and 90%, with protection against severe cases and ICU admissions ranging from 75 to 85%.^149^, ^150^, ^151^ Furthermore, nirsevimab has received regulatory clearance in the European Union, the United Kingdom, and China, positioning it as a preventative measure for RSV infection in healthy term infants, preterm infants, and infants predisposed to severe RSV infection owing to specific health conditions.^152^

#### 3.2.3 Clesrovimab (MK‐1654)

Clesrovimab, a fully human neutralizing mAb against RSV developed by Merck, incorporates YTE mutations that augment its half‐life and targets the site IV of the F protein.^153^ In vitro research has illustrated the efficacy of clesrovimab against both RSV subtypes A and B, showcasing its capacity to effectively neutralize a spectrum of clinical isolates.^137^ The safety and immunogenicity profiles of clesrovimab in healthy adults are commendable.^154^, ^155^ In July 2024, Merck reported positive outcomes from the Phase 2b/3 clinical trial MK‐1654‐004 (NCT04767373) investigating the preventative potential of clesrovimab against RSV infection. This trial was a randomized, double‐blind, placebo‐controlled study that evaluated the efficacy and safety of a single dose of clesrovimab administered via intramuscular injection to protect infants, including both healthy preterm and term neonates, from RSV infection. The findings revealed that the trial achieved its primary endpoints concerning safety and efficacy, notably the reduction in the occurrence of medically attended lower respiratory tract infections precipitated by RSV within a 150‐day period, suggesting its potential as a robust preventative intervention for RSV infections in the future.

### 3.3 Unresolved issues and remaining challenges of RSV antibody

In 2022, nirsevimab gained regulatory approval and was successively marketed in the European Union, the United Kingdom, the United States, and China, addressing the challenge that palivizumab could only be used for the prevention of RSV infection in high‐risk infants and young children. It can now be utilized for the prevention of RSV infection in all infants and young children, marking a significant advancement in the passive immunoprophylaxis against RSV infection in this demographic. However, the development of RSV mAbs has not reached its terminus, with numerous unresolved issues and challenges remaining: (1) insufficient monitoring of virus evolution data and unknown impact on mAb escape, (2) no marketed therapeutic RSV mAbs, (3) effects of antidrug antibodies (ADAs) need to be assessed, (4) the interference of RSV mAb administration with existing vaccination schedules in different countries requires investigation, (5) the coexistence of preventive strategies such as RSV maternal vaccines, passive immunoprophylactic antibodies, and vaccines in development for infants and young children warrants further research, and (6) accessibility and affordability of RSV mAbs in LMICs.

#### 3.3.1 Insufficient monitoring of virus evolution data and unknown impact on RSV antibody escape

RSV bears a significant global disease burden, leading to complex characteristics of genetic evolution, phylogeny, and geographical distribution.^7^, ^156^, ^157^ Surveillance and tracking of viral mutations, infection profiles across different age groups, epidemiological conditions, and disease burdens hold substantial scientific and practical value for disease prevention and early warning, evaluation of vaccine and mAb efficacy, and analysis of socio‐economic impacts.^158^, ^159^ Therefore, continuous monitoring of viral evolution and genetic surveillance is of paramount importance, and there is an urgent need to establish a global RSV monitoring and information system. Currently, the Global Initiative on Sharing All Influenza Data (GISAID) has preliminarily established a global RSV monitoring system based on the Global Influenza Surveillance and Response System, which serves to understand the global spread of the virus, risk factors for RSV infection and severe disease. GISAID provides RSV genetic sequences, epidemiological data, reference sequences for different RSV subtypes, phylogenetic trees for various regions, and monitors mutations in the RSV F protein.^160^, ^161^ Additionally, the ReSViNET Foundation, the first nonprofit organization globally dedicated to mitigating the burden of RSV, has developed the world's first RSV monitoring dashboard, tracking seasonal variations of RSV globally, including at least 20 countries across different geographical areas of both hemispheres.^162^ However, the current global monitoring network and data still require refinement, especially in LMICs, making the establishment of a standardized, dynamic, and public RSV infectious disease monitoring system extremely urgent.

The development of viral drug resistance is particularly relevant to the immunoprophylaxis of infants with mAbs. It is well recognized that mAbs for passive immunoprophylaxis against RSV bind to the RSV F protein, thereby preventing viral entry into and replication within the host cells. Consequently, mAbs are exceptionally sensitive to viral mutations; a mutation at a single site in the protein may result in complete immune evasion of the virus, leading to the emergence of drug resistance.^163^, ^164^ Suptavumab (REGN2222), an RSV mAb targeting the site V of the RSV F protein, is a fully human IgG1 mAb with more than fivefold increased affinity compared with palivizumab. However, the results of the Phase 3 clinical trial indicated that the efficacy of suptavumab in preventing RSV infection did not meet the primary endpoints. Analysis revealed that the antigenic sites at positions 172 and 173, recognized by suptavumab, underwent mutations in circulating strains, particularly in the RSV B subtype, leading to a reduction in neutralizing activity and even ineffectiveness of suptavumab, ultimately resulting in the discontinuation of its development.^165^ Of greater concern is that mutations will continue to emerge during the circulation of the RSV virus, and whether these mutations will affect the neutralizing activity of marketed antibodies, especially nirsevimab targeting the site Ø, requires ongoing surveillance.^166^, ^167^ Moreover, the combined use of mAbs targeting different sites could be considered to prevent the emergence of viral drug resistance. Regrettably, to date, no cocktail regimen involving the combined use of multiple mAbs has been employed for RSV passive immunoprophylaxis.

#### 3.3.2 No marketed therapeutic RSV mAbs

Currently, maternal vaccines and the mAb product nirsevimab have been utilized for the prevention of RSV infection in infants and young children, offering substantial protection to susceptible populations against RSV.^168^ However, after RSV infection in infants and young children, there is still a lack of effective specific therapeutic agents or mAbs. After palivizumab was approved for preventing severe RSV disease in specific high‐risk groups, a double‐blind, randomized controlled trial was conducted to assess its efficacy in treating acute RSV infection. The results indicated no significant differences between the palivizumab group and the placebo group in terms of hospitalization rates, duration of hospital stay, and admission rates to the Pediatric Intensive Care Unit.^169^ Apart from that, motavizumab is a second‐generation humanized mAb against RSV. A multicenter, randomized controlled trial studied the impact of motavizumab on pediatric patients who tested positive for RSV, and the results also showed no differences between the motavizumab treatment group and the placebo control group in terms of hospital stay duration, illness severity, or subsequent wheezing episodes.^170^ Additionally, while nirsevimab has shown excellent prophylactic effects, there were no significant differences when compared with the placebo group in terms of discharge time after hospitalization for RSV infection in children. Moreover, ALX‐0171, an inhaled nanobody under development by Ablynx, a subsidiary of Sanofi, is a nanobody trimer targeting site II. Clinical trials have shown that, compared with placebo, inhaled ALX‐0171 can reduce viral load and directly affect RSV replication, but there was no difference between the treatment and placebo groups based on the primary endpoint of discharge time.^171^ The reasons for the clinical failure of RSV therapeutic mAbs are unclear and are presumably related to the fact that most of the patients enrolled in the clinical trials were children who sought medical attention only after they had already developed severe symptoms. In addition, the long time it takes for the mAb to reach the lungs and exert antiviral efficacy after administration may be another important reason for clinical failure of RSV therapeutic mAbs. In summary, the researchers hypothesized that delivery of mAbs to the lungs by nebulized inhalation methods in the early stages of RSV infection may be a potential therapeutic approach for acute RSV infection,^172^, ^173^ but to date, no RSV therapeutic mAbs have achieved excellent clinical trial results.

#### 3.3.3 Assessment of the effects of ADAs

RSV mAb products possess well‐defined targets and specific mechanisms of action, which contribute to their improved safety and efficacy profiles. However, antibodies may also be recognized as foreign entities by the host, potentially triggering undesirable immune responses that can lead to the production of ADAs. Initially, ADAs were thought to be associated with the murine sequences present in antibodies, which are recognized by the human immune system, such as palivizumab, which retains approximately 5% murine sequence. Nevertheless, the presence of a complete human antibody gene has not eliminated immunogenicity and the associated occurrence of ADAs. This may be due to novel sites in the complementarity‐determining regions of fully human mAbs. In clinical trials for palivizumab, motavizumab, and nirsevimab, not only were the safety, efficacy, and PK of the mAbs assessed, but also the antibody tolerance and the presence of ADAs were evaluated. The detection rate of ADAs ranged from 3 to 28%, with no correlation found between ADAs and adverse reactions.^174^, ^175^, ^176^, ^177^ However, with the increasing number of mAbs marketed for various conditions, including cancer, chronic autoimmune diseases, inflammatory diseases, allergies, and infections, the use of mAbs in more than one dose will become more common. Therefore, it is essential to continue evaluating the development and impact of ADAs for RSV antibodies.

#### 3.3.4 Investigation of RSV mAbs impact on vaccination programs in different countries

The administration of RSV mAbs necessitates a comprehensive consideration of various factors, including the age of infants and young children, the time of birth, and the RSV epidemic seasons in different countries, thereby creating vaccination needs for children of different months of age. Each country has its existing recommended vaccination schedules and immunization procedures, and the use of RSV mAbs may interfere with the efficacy of certain vaccines. Similarly, there is a phenomenon where the existing vaccination schedules and immunization procedures in different countries may affect the passive immunoprophylactic effects of RSV mAbs. However, there is a paucity of research regarding the interference between RSV mAb injections and existing vaccination schedules. Therefore, it is recommended that postmarketing surveillance of RSV mAbs be conducted in different countries to ensure the efficacy of both the RSV mAbs and other vaccines.^178^

#### 3.3.5 Coexistence of RSV preventive strategies: maternal vaccines, passive immunoprophylactic antibodies, and infant/young child vaccines in development

As infant immunoprophylactic agents and maternal vaccines become commercially available and with the ongoing advancement of clinical trials for infant and children's vaccines, the interaction and concurrent use of RSV preventative measures will inevitably raise critical issues.^179^ Maternal immunization has the capacity to increase the levels of RSV‐specific antibodies in expectant mothers, facilitating their transfer across the placenta and thereby circumventing the necessity for direct neonatal immunization. Nonetheless, maternal vaccines confront significant challenges, such as the diminished antibody transfer in preterm infants and the potential inadequacy of the protective duration of maternal antibodies secured through placental transfer, which may not span the entire RSV epidemic season as a consequence of the decline in maternal antibody titers postpartum. Consequently, passive immunization via mAbs can offer safeguarding to infants under the age of 2 years whose mothers have either not received vaccination or whose maternally derived antibodies are inadequate for conferring protection. Furthermore, vaccines intended for infants and children are currently in Phase 3 clinical research, holding the promise of extending protection to the pediatric population above 6 months of age. However, the coexistence or the recommended sequence of employment for the preventive strategies remains a subject that warrants further investigation.^180^, ^181^ Therefore, the design of future clinical trials for RSV preventative products must carefully consider the complex prevention needs of diverse populations, requiring more nuanced trial designs tailored to different age groups, risk categories, and product types.

#### 3.3.6 Accessibility and affordability of RSV mAbs in LMICs

RSV is the leading cause of acute LRTI in children under the age of two worldwide, with approximately 97% of infant deaths attributed to RSV infections occurring in LMICs. Moreover, pregnant women in LMICs are significantly less likely to receive vaccinations than those in developed countries, increasing the reliance on mAbs for RSV prevention in infants and young children. Therefore, for global RSV prevention efforts, priority consideration must be given to the accessibility and affordability of interventions in LMICs. Furthermore, there is a need to expedite evaluations of the cost‐effectiveness of RSV prevention strategies, the correlates of RSV protection, and the efficacy of RSV prevention measures in these countries.^182^ Of paramount importance is the enhancement of international collaboration and resource sharing to reduce the economic costs of RSV mAbs in LMICs and to ensure their prioritized supply.

## 4 RSV THERAPEUTIC AGENTS

The therapeutic objective during the acute phase of RSV infection is to alleviate symptoms, diminish viral replication and duration, mitigate the severity of the disease, and reduce the risk of transmission. To date, there is no specific and effective treatment for RSV that is globally recognized. As more insights are gleaned regarding the mechanisms of RSV infection, a multitude of innovative drugs are currently in the clinical development phase worldwide, with the potential to achieve groundbreaking advancements.

### 4.1 Progress in RSV therapeutic agents research and development

**FIGURE 4:**
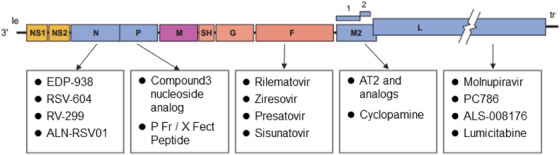
RSV viral protein targets and respective therapeutic agents. N, nucleoprotein; P, phosphoprotein; F, fusion protein; M2‐1 protein, transcription factor; L, large viral polymerase.

Therapeutic agents for RSV include both specific and nonspecific drug categories.^183^, ^184^, ^185^, ^186^ Nonspecific drugs currently constitute the primary modality of treatment, managing acute RSV LRTI through supportive care and symptomatic relief. Supportive treatment includes timely rehydration, nutritional support, maintenance of airway patency, and oxygen therapy. Symptomatic treatment often employs mucolytics, bronchodilators, and anti‐inflammatory agents to reverse airway obstruction and alleviate symptoms of infection. Commonly utilized medications include leukotriene receptor antagonists, salbutamol, N‐acetylcysteine, corticosteroids, nebulized hypertonic saline, epinephrine, and antibiotics.^25^, ^187^ The following discussion will focus on elucidating specific RSV therapeutic agents (Figure 4 and Table 2).

**TABLE 2:** The research and development progress of RSV therapeutic agents.

Protein targets of therapeutic agents	Name	Modality	Company/institute	Current development stage
Host IMPDH inhibitors	Ribavirin (Rebetol)	SM	Merck, Viratek (Valeant)	Launched
RSV fusion protein inhibitors	Rilematovir (JNJ‐53718678)	SM	Johnson & Johnson	Discontinued
	Ziresovir (AK‐0529)	SM	Ark Biosciences, Roche	Preregistration in China
	Presatovir (GS‐5806)	SM	Gilead	Discontinued
	Sisunatovir (RV521)	SM	Pfizer (Reviral previously)	Phase 2
	BMS‐433771	SM	Bristol Myers Squibb	Discontinued
	BTA‐9881 (AZD‐9639, MEDI‐564)	SM	Spark (previously Aviragen, previously Biota)	Discontinued
	TMC‐353121 (R‐391036, JNJ‐27387581)	SM	Johnson & Johnson	Discontinued
RSV large polymerase protein inhibitors	PC786	SM	Pulmocide	Discontinued
	Lumicitabine (ALS‐8176, JNJ‐64041575)	SM	Johnson & Johnson (previously Alios Biopharma)	Discontinued
RSV nucleoprotein inhibitors	EDP‐938 (EP‐023938)	SM	Enanta	Phase 2
	RSV‐604 (A‐60444)	SM	AstraZeneca (previously Arrow Therapeutics)	Discontinued
	RV‐299 (PF‐07923567)	SM	Pfizer (previously ReViral)	Discontinued
	ALN‐RSV01	siRNA	Alnylam	Discontinued
RSV M2‐1 inhibitors	AT2 and analogs	SM	Virion Systems, Cardiff University/KUL	Not reported
	Cyclopamine	SM	CombinatoRx, multiuniversity	Not reported
RSV phosphoprotein inhibitors	Compound 3	SM	AstraZeneca	Discontinued
	P Fr / X Fect	Peptide	Kurume University School of Medicine, Japan and CNRS/INRAE	Not reported

Table 2 provides publicly information of RSV therapeutic agents. Sources of information for these trials are the website https://clinicaltrials.gov/, and investigators, sponsors, funders of clinical trials.

Abbreviations: SM, small molecule, siRNA, small interfering RNA.

#### 4.1.1 Ribavirin

Ribavirin is a guanosine nucleotide analog that inhibits viral proliferation by disrupting viral synthesis. It is a widely used broad‐spectrum antiviral medication, applicable for the treatment of a variety of viral diseases and is currently the only antiviral drug approved for the treatment of viral pneumonia and bronchiolitis caused by RSV in infants and children. When administered early in the course of the disease, ribavirin can prevent the progression from upper respiratory tract infection to LRTI in patients. Ribavirin is available in three dosage forms: inhalation, oral, and intravenous.^188^, ^189^ In 1985, inhaled ribavirin was approved by the US FDA for marketing, indicated for the treatment of hospitalized children with severe LRTI caused by RSV. In our country, both oral and parenteral administration have been approved for the treatment of RSV. However, due to ongoing debates regarding the safety and efficacy of ribavirin, it is currently recommended only for severe disease and complications, and for immunocompromised or immunodeficient infants and children at high risk of RSV infection.^41^, ^42^, ^190^

#### 4.1.2 Inhibitor of RSV fusion protein

Inhibitors of the F protein function as fusion inhibitors, intervening at the critical juncture of virus‐host cell binding and subsequent invasion. These inhibitors achieve their therapeutic effect by complexing with the F protein of RSV, thereby impeding the conventional fusion of the viral envelope with the host cell membrane. This interaction precludes the virus from entering the cell, thus serving as a therapeutic intervention for RSV infection. At present, a suite of fusion inhibitors has advanced into clinical development and investigative stages, comprising rilematovir (JNJ‐53718678), ziresovir (AK‐0529), presatovir (GS‐5806), sisunatovir (RV521), and several additional candidates.

Rilematovir (JNJ‐53718678), a therapeutic agent developed by Janssen, has shown in Phase 2a clinical trials that its early administration may confer potential clinical advantages for adults afflicted with RSV infection.^191^ However, a separate Phase 3 clinical trial evaluating the efficacy of rilematovir in treating infants and neonates hospitalized with ARIs caused by RSV was discontinued before completion.^192^

Ziresovir (AK‐0529), developed by ArcBio, exhibits rapid onset of action and good compliance, demonstrating more pronounced clinical benefits in infants under 6 months of age, along with a favorable safety and tolerability profile.^193^ Currently, Phase 3 clinical trials have been completed; however, the marketing authorization application for ziresovir enteric‐coated capsules has not been approved by China's Center for Drug Evaluation.

Presatovir (GS‐5806), developed by Gilead Sciences, has demonstrated in Phase 1 clinical trials the ability to reduce RSV viral load and disease severity in healthy adults, exhibiting favorable safety and PK profiles.^194^ However, in a clinical trial involving hematopoietic stem cell transplant recipients with RSV‐associated upper and lower respiratory tract infections, presatovir, while showing good safety and tolerability, did not improve virological or clinical outcomes and failed to meet the primary study endpoints.^195^, ^196^ Furthermore, in a Phase 2b clinical trial among adults with RSV infection following lung transplantation, presatovir also did not significantly improve patient RSV load, symptoms, or lung function.^197^ Currently, there are no Phase 2 or III clinical trials for presatovir in pediatric populations.

Sisunatovir (RV521), developed by ReViral, has demonstrated exceptional safety and therapeutic metrics in preclinical trials and Phase 1 clinical trials. In a Phase 2a RSV human challenge study, sisunatovir significantly reduced viral load and clinical symptoms.^198^, ^199^ Currently, a global multicenter, randomized, double‐blind, interventional Phase 2/3 study is underway to investigate the efficacy and safety of oral sisunatovir in the treatment of nonhospitalized adult patients with RSV infection who are at risk of progressing to severe disease.

#### 4.1.3 Inhibitor of RSV large polymerase protein

The RSV L protein inhibitor is a replication inhibitor that functions by suppressing the activity of the RSV polymerase, thereby halting the replication of the viral genomic RNA and the transcription of mRNA. This action prevents the effective replication of the virus, making it a therapeutic agent for the treatment of RSV infection. Currently, replication inhibitors that have entered the clinical development and research phases include PC786, ALS‐008176, and Lumicitabine (JNJ‐64041575).

PC786, developed by Pulmocide Ltd, is a nebulized non‐nucleoside RSV polymerase inhibitor that achieves therapeutic efficacy by inhibiting RSV replication on the surface of respiratory epithelial cells.^200^, ^201^ A Phase 1/2 clinical study was conducted to assess the antiviral activity, safety, and PK of multiple dosing of PC786. The results indicated that nebulized PC786 exhibits significant antiviral activity against RSV.^202^ However, the Phase 2 clinical trial is currently in a suspended state, which may suggest adjustments and obstacles encountered during the clinical development process.

ALS‐008176, developed by Alios Biopharma Inc., is a novel inhibitor of RSV replication, functioning as a triphosphate nucleotide analogue to suppress the replication of RSV. Multiple clinical trials have demonstrated that ALS‐008176 can reduce the viral load, the area under the curve of viral load, the peak viral load, and the duration of viral shedding in subjects infected with RSV.^203^ However, this drug has not yet progressed to Phase 3 clinical trials. It was later continued to be developed by Johnson & Johnson and renamed lumicitabine (JNJ‐64041575).^204^ Phase 1b and Phase 2 clinical studies evaluated the safety, PK, and pharmacodynamics (PD) of lumicitabine in infants and neonates hospitalized due to RSV infection. The results indicated no significant differences between the lumicitabine group and the placebo group in terms of reducing viral load, the interval to achieve undetectable viral levels, and symptom alleviation. Lumicitabine failed to demonstrate antiviral activity in hospitalized infants infected with RSV and was associated with dose‐related neutropenia.^205^ Currently, multiple Phase 2 clinical trials of this drug have been terminated, signaling the failure of drug development.

### 4.2 Unresolved issues and remaining challenges of RSV therapeutic agents

In recent years, there have been breakthrough advancements in the development of RSV vaccines and passive immunization mAbs. However, the progress in the development of RSV therapeutic agents has not met expectations, with no new specific drugs being marketed. The development of therapeutic products for the treatment of acute RSV infection still faces numerous bottlenecks and challenges, including: (1) the efficacy of drugs targeting viral protein targets remains to be elucidated, (2) the feasibility of drugs targeting host proteins necessitates thorough evaluation, (3) the divergences observed between preclinical studies and clinical trials for therapeutic agents must be addressed, (4) the variations in clinical trials of therapeutic agents among diverse patient populations need to be delineated, (5) the potential risk for drug resistance in RSV therapeutic agents warrants close attention.

#### 4.2.1 Efficacy of drugs targeting viral protein targets remains to be elucidated

The development of antiviral agents against RSV faces numerous challenges, and ribavirin remains the only pharmaceutical agent approved for treating acute RSV infections. To date, at least 30 drug candidates targeting viral proteins have been discontinued or have failed in development. Current research for RSV therapeutics is concentrated on inhibiting the F protein to prevent viral entry and the viral RNA‐dependent RNA polymerase (L protein) to suppress viral replication, as previously delineated. Moreover, therapeutics targeting additional viral proteins such as the N protein, M2‐1 protein, P protein, SH protein, and NS1 protein are under investigation for the treatment of acute RSV infections, aiming to impede viral genome replication, transcription, trafficking, and release. Notably, N protein inhibitors that have entered clinical trials include EDP‐938 (EP‐023938),^119^ RSV‐604 (A‐60444),^206^ RV‐299 (PF‐07923567), and ALN‐RSV01.^207^ Presently, aside from EDP‐938 which is in Phase 2 clinical trials, the development of the other three drugs has been discontinued. The M2‐1 protein, crucial for viral transcription, has emerged as a target for the treatment of acute RSV infections. In this regard, AT2 and its analogs act as inhibitors by displacing the central Zn^2+^ cation of the tetramer, thereby exerting antiviral effects; Cyclopamine functions by hardening inclusion bodies and blocking the M2‐1/P interaction.^208^ The P protein, indispensable for viral transcription and replication, is also a target for RSV infection therapy. AstraZeneca has identified cpd 3 nucleoside analog inhibitors through high‐throughput screening from approximately one million compounds, which induce a D231 V mutation in the P protein to elicit antiviral activity.^209^ The P Fr/X Fect Peptide, a fragment of the P protein's C‐terminal region encompassing residues 130–180 and 212–241, inhibits viral infection by preventing oligomerization and interaction with N.^210^ Collectively, the development of novel RSV therapeutics targeting viral proteins is progressing, albeit with numerous studies previously halted due to issues of safety and clinical efficacy. Consequently, there is an ongoing need for extensive research and investment by the scientific community and industry to further investigate the efficacy and safety profiles of drugs targeting various viral proteins.

#### 4.2.2 Feasibility of host protein‐targeted drugs requires thorough evaluation

The disease severity ensuing from RSV infection is associated not only with the vigor of viral replication but is predominantly modulated by the host immune response. Upon infection, RSV incites the host's immune system, leading to the activation and release of inflammatory mediators that are instrumental in viral clearance but may also precipitate immune dysregulation and subsequent immunopathology. Host factors, including chemokine receptor CCR1,^211^ chemokine (C‐C motif) ligand 2, retinoic acid receptor responder protein 2, ephrin‐B2,^212^ mTOR kinase,^213^ nuclear export protein exportin 1 (XPO1), nucleolar protein nucleolin (NCL), Toll‐like receptor 4, intercellular adhesion molecule‐1, leukotrienes, and host inosine‐5′‐monophosphate dehydrogenase (IMPDH), serve as receptors, coreceptors, and inflammatory mediators essential for RSV entry and replication within host cells. The development of RSV therapeutics targeting host factors is garnering escalating interest. Verdinexor (KPT‐335), a selective inhibitor of nuclear export, has demonstrated the capacity to diminish RSV replication in vitro.^214^ AS1411, an anticancer compound that engages with NCL on the cell surface, has been shown to partially attenuate RSV lung viral titers and mitigate virus‐associated airway inflammation in murine and cotton rat models.^215^ Novel antiviral agents that target host factors can potentially curtail RSV replication and ameliorate the inflammatory response induced by RSV infection. Nonetheless, the enhancement of their nonspecific antiviral potency and a thorough investigation into their potential toxicity are areas that require further exploration.

#### 4.2.3 Addressing divergences between preclinical studies and clinical trials of RSV therapeutic agents

A multitude of therapeutic agents that have demonstrated exceptional performance in preclinical studies have met with failure in clinical trials. The main reason for these setbacks is that preclinical studies often fail to adequately reflect the therapeutic efficacy and safety profiles of drugs in real‐world scenarios. Secondary causes may be due to deficiencies in clinical trial protocols. Presatovir (GS‐5806), developed by Gilead Sciences following high‐throughput antiviral screening and chemical optimization, exhibits selective inhibition against 75 clinical isolates of RSV subtypes A and B, with an average effective concentration (EC50) of 0.43  nM, classifying it as an efficacious RSV fusion inhibitor.^216^ Furthermore, a randomized, double‐blind, placebo‐controlled study of oral presatovir in healthy adults challenged with a clinical strain of RSV via the intranasal route indicated that treatment with presatovir resulted in a reduction of viral load and clinical disease severity.^194^ However, Phase 2 clinical trials of presatovir in recipients of hematopoietic stem cell transplants^196^ and lung transplants^197^ did not achieve significant improvements in patient viral loads or symptomatology, failing to attain the primary study endpoints and culminating in failure. The analysis of the reasons for the failure of these clinical trials suggests that early treatment was administered before symptom onset in the challenge trial, while treatment was initiated only after symptom onset in the other two clinical trials. Additionally, several compounds, including BTA‐9881 (AZD‐9639, MEDI‐564) and TMC‐353121 (R‐391036, JNJ‐27387581), displayed promising antiviral activity in preclinical studies but were associated with safety concerns in clinical trials.^186^ Moreover, numerous drug candidates have failed due to low clinical trial enrollment rates, shifts in RSV prevalence seasons, variability among enrolled patients, and issues related to establishing primary endpoints, all of which indicate clinical trial design challenges.^186^ In conclusion, the design of clinical trials for RSV therapeutic agents is of paramount importance. Concurrently, during the preclinical research phase, it is imperative to consider the real‐world dynamics of viral circulation and medication usage to mitigate the risk of clinical trial failures and to avert the squandering of substantial research and development expenditures.

#### 4.2.4 Analyzing variations in therapeutic agent clinical trials among diverse patient populations

Lumicitabine (JNJ‐64041575) is an oral nucleoside analogue that was evaluated in a human challenge study with RSV type A virus to assess the kinetics of RSV infection and the PK and PD of lumicitabine in adults by monitoring viral load and drug metabolite levels. The clinical trial outcomes suggested that lumicitabine has antiviral efficacy.^204^ Conversely, a Phase 1b and 2b clinical trial investigating the use of lumicitabine in hospitalized infants infected with RSV indicated that the drug did not demonstrate significant antiviral activity in terms of reducing viral load or ameliorating infection symptoms, and it led to an adverse effect of neutropenia in a dose‐dependent manner.^205^ The disparity in the therapeutic effects of lumicitabine between infants and adults may stem from several factors: first, the presence of preexisting immune memory against RSV in the adult population; second, the fact that infections in adult challenge trials are predominantly localized to the upper respiratory tract. These differences in immune profiles and the severity of clinical disease across populations have resulted in variances in the drug's efficacy and safety profiles. Therefore, the design of clinical trials for RSV therapeutics should meticulously consider the distinct characteristics of different RSV‐affected populations, and it is imperative to devise clinical trial protocols and clinical observation endpoints that are well reasoned and appropriate.

#### 4.2.5 Drug resistance in RSV therapeutic agents warrants attention

Therapy with antiviral medications poses the risk of inducing drug resistance, as evidenced by resistance development against amantadine in influenza viruses; hence, there is an analogous risk of resistance to RSV therapeutic agents. In an assessment of resistance to the RSV F protein inhibitor PRESATOVIR, it was observed that 28 mutations at F protein sites emerged during treatment, with 16 of these mutations leading to a reduction in RSV sensitivity to presatovir by a factor of 2.9–410 times. Additionally, lower dosages of presatovir and shorter durations of treatment were correlated with an increased frequency of resistant mutations. Fortunately, despite the emergence of resistant RSV strains following presatovir administration, there was no significant impact on the drug's clinical efficacy.^217^, ^218^ A study investigating the combination of antiviral drugs with distinct mechanisms against RSV revealed that the coadministration of fusion inhibitors (GS5806, ziresovir, and BMS433771) and RdRp inhibitors (ALS8176, RSV604, and Cyclopamine) demonstrated synergistic antiviral activity, whereas antagonistic interactions were noted among the fusion inhibitors.^219^ Consequently, the concomitant use of RSV fusion inhibitors and replication inhibitors has the potential to optimize therapeutic efficacy and mitigate the risk of resistance, underscoring the urgent need for further validation through animal models and clinical trials.

## 5 CONCLUSIONS

Over the course of six decades of research, breakthroughs have been made in RSV vaccines, mAbs, and therapeutic agents. Particularly noteworthy is the elucidation of the RSV PreF protein structure and antigenic sites, which has laid the groundwork for the precise design and successful market introduction of vaccines and mAbs. Currently, three prophylactic RSV vaccines are available: Arexvy, Abrysvo, and mRESVIA, but there are still unresolved difficulties and challenges. The most critical challenge is that there is no vaccine for the pediatric population older than 6 months of age. In addition, safety issues such as the risk of GBS after vaccination in the elderly population and the risk of preterm labor due to the vaccine in pregnant women need to be further evaluated. Furthermore, the RSV vaccine has just been approved in 2023 and has already been found to be less effective during the second RSV epidemic season. Therefore, further data on the long‐term protective properties of the vaccine need to be collected, and it is hypothesized that multiple doses of the RSV vaccine may be required. Furthermore, RSV vaccines rely on immune homeostasis, and the use and selection of adjuvants is inconclusive. Meanwhile, RSV multiplex and multivalent vaccines are still in the early stages of development. In summary, RSV vaccines still face multiple challenges at different stages of early development, preclinical studies and clinical trials.

Beyond RSV vaccines, passive immunoprophylactic antibodies have also achieved significant progress. The long‐acting mAb nirsevimab has been granted regulatory approval for the prevention of RSV infection in healthy term infants, preterm infants, and infants with specific health conditions that confer an increased risk of severe RSV infection. Nonetheless, RSV passive immunoprophylactic mAbs confront numerous challenges. First, RSV evolution data are insufficiently monitored, and the impact of mAb escape due to mutation is unknown. More urgently, there is a lack of effective and specific therapeutic mAbs for RSV infection in infants and children. In addition, the potential ADAs effect and the interference with existing vaccination schedules in different countries remain to be examined. Another key issue is that the coexistence of prevention strategies such as RSV maternal vaccines, passive immunoprophylactic mAbs, and vaccines under development for infants and young children remains further investigation.

More alarmingly, there are currently no approved antiviral therapeutic agents for RSV, with treatment options largely limited to symptomatic and supportive care. In particular, the failure of several therapeutic agents targeting viral proteins has increased the difficulty of antiviral drug development. In addition, the effectiveness and safety issues of drugs targeting host proteins still need to be further investigated. Furthermore, the clinical trials of several therapeutic agents with excellent preclinical results ended in failure, which also put forward higher requirements for the design of clinical trials. Additionally, the population efficacy differences of RSV antiviral drugs and the risk of drug resistance need to be further evaluated. Currently, the counter‐seasonal prevalence and atypical scale of RSV infections introduce considerable uncertainties for the development of specific RSV therapeutics.^220^, ^221^ Consequently, there is an urgent need to employ novel strategies and technologies in medicinal chemistry, as well as drug repositioning, to explore synergistic multitarget and multimechanism drug therapies, thereby enhancing the efficacy and clinical success rates of the next generation of RSV therapeutic agents.^222^ Given the failure of numerous clinical trials targeting viral protein, the timeline for the successful market introduction of RSV therapeutic agents remains uncertain. Therefore, while expediting the development of specific RSV therapeutic agents, it is imperative to enhance the accessibility of preventive mAbs and vaccines through the rational allocation of medical resources to mitigate the disease burden imposed by RSV infections.

## AUTHOR CONTRIBUTIONS

*Conceptualization*: Youchun Wang and Qianqian Li. *Methodology*: Qianqian Li. *Formal analysis*: Huan Li. *Writing—original draft preparation*: Qianqian Li. *Writing—review and editing*: Qianqian Li, Huan Li, Zhihua Li, and Youchun Wang. *Supervision*: Youchun Wang. All authors have read and agreed to the final manuscript.

## CONFLICT OF INTEREST STATEMENT

The authors declare no conflict of interest.

## ETHICS STATEMENT

Not applicable.

## Data Availability

Data sharing is not applicable.
